# Clinical exome sequencing in France and Quebec: what are the challenges? What does the future hold?

**DOI:** 10.1186/s40504-018-0081-2

**Published:** 2018-08-01

**Authors:** Gabrielle Bertier, Yann Joly

**Affiliations:** 1 Department of Human Genetics, Center of Genomics and Policy, McGill University, 740 Dr. Penfield Avenue, Montreal, Quebec H3A 0G1 Canada; 2 Université Toulouse III Paul Sabatier and Inserm UMR 1027, 37 allées Jules Guesde, F-31000 Toulouse, France

**Keywords:** Multiple case study, Next-generation sequencing, Whole-exome sequencing, Rare diseases, Cancer genetics, France and Quebec, Healthcare systems, Health policy

## Abstract

**Background:**

The decreasing cost of next-generation sequencing technologies (NGS) has resulted in their increased use in research, and in the clinic. However, France and Quebec have not yet implemented nation-wide personalized medicine programs using NGS. To produce policies on the large-scale implementation of NGS, decision makers could benefit from a detailed understanding of how these technologies are currently used, their limitations, and the benefits they could bring to patients.

**Objectives:**

We aimed at answering two research questions: How are patients’ NGS data currently managed in healthcare institutions in Quebec and in France? What issues do technology users identify which should be solved in order to implement clinical genomics at the national level?

**Method:**

Through a multiple case study method, we analysed interviews and documentation from four teams that use whole-exome sequencing in hybrid clinical research projects focusing on cancer and rare diseases.

**Results:**

Interviewees detailed numerous challenges linked with managing the complexity of the process of collecting and interpreting data in a relevant manner for patients, and described how obtaining buy-in from multiple stakeholders was necessary.

**Conclusion:**

A strong political will is essential for personalized medicine to be implemented efficiently in France and Quebec.

**Electronic supplementary material:**

The online version of this article (10.1186/s40504-018-0081-2) contains supplementary material, which is available to authorized users.

## Introduction

The decreasing cost of human genome sequencing technologies (National Human Genome Research Institute (NHGRI) 2015) has resulted in their increased use in research and in the clinic(Steinbock and Radenovic 2015). Indeed, next-generation sequencing (NGS) research results have proven that the correct interpretation of a human genome can improve diagnostic yield for rare diseases(Chérot et al. 2018; Hartley et al. 2018; Lubitz and Ellinor 2015; Tan et al. 2017b), and enable a greater efficacy of treatment of certain cancers(Dieci et al. 2016; Harris et al. 2016; Hintzsche et al. 2016; Parsons et al. 2016; Ramkissoon et al. 2017; Tan et al. 2017a). Recently, the use of these technologies in neonatal care especially in critically ill infants has been launched with great promise and some controversy(Borghesi et al. 2017; Char 2015; Meng et al. 2017; Reardon 2014; Smith et al. 2016). Today, the sequencing and analysis of a patient’s whole exome or whole genome is offered to specific patient groups in a limited number of health institutions around the world, such as in the USA(Green et al. 2016; Lee et al. 2014; Lionel et al. 2017; Swaminathan et al. 2017) or in the Netherlands(van Zelst-Stams et al. 2014),or in some other developed countries, in the context of pilot or proof-of-concept projects(Hartley et al. 2018; Lacroix et al. 2014; Lefebvre et al. 2015; Parsons et al. 2016). In the UK, the Public Health Genomics Foundation has published a number of technical(Finnegan and Hall 2017; Luheshi Leila and Sobia 2014; Raza 2014) and policy reports (Alison Hall et al. 2014; Burton 2015) in order to accompany the progressive use of genomic sequencing technologies in « mainstream clinical pathways»(Burton et al. 2017) in the country, a topic which has generated discussions at the national level (Bourn 2017; Davies 2016). The UK’s 100.000 genomes project, as well as the United States’ precision medicine initiative, renamed the “all-of-us research program”, as well as, are two examples of large-scale national initiatives in which governments have invested significant resources to build an infrastructure enabling the clinical use of NGS. In this study, we focused on two jurisdictions which have not yet publicly embarked on endeavours of a comparable scale: Quebec and France.

The clinical implementation of NGS poses a number of challenges(Bertier et al. 2016b), especially in pediatric populations(Bertier et al. 2017). Several steps must be performed to enable the data to be transformed, from a raw sequence, to a clinically informative report readable by a physician. However, costs are still high(Weymann et al. 2017), most of the data is still difficult to interpret(Ghazani et al. 2017), and bioinformatics tools and pipelines, and data interpretation strategies are only partially standardized at the moment. To be able to produce policy on the large-scale implementation of NGS, decision makers need to understand what this process of standardization entails, and how it currently unfolds within the scientific and clinical genomics communities. Although numerous teams publish the results they obtain with clinical genomics projects, no case study has been published to our knowledge detailing how French or Quebec teams operate and how those projects function in detail. In this study, we aimed at answering the following research questions: How are patients’ NGS data currently managed (produced, accessed, analysed, interpreted and shared) in specific healthcare institutions in Quebec and in France? What issues do technology users identify which should be solved in order to implement clinical genomics at the national level? To answer this question, we used a multiple case study research method.

## Materials and methods

Case studies research is particularly adapted to the study of complex contemporary phenomena(Mucchielli 2004). The phenomenon under examination here is the clinical use of NGS. The small number of teams using these technologies in patient care in France and Quebec, as well as the rapid pace at which these technologies are currently developing makes case studies an appropriate methodology to study this phenomenon. We followed the multiple embedded case study methodology as described by Robert Yin(Yin 2008).

To select cases, we looked for teams which were using NGS to inform patient care, in the context of comparable projects in France and in Quebec. There were two main reasons for us to focus on these two jurisdictions: First, although they have not embarked in large scale precision medicine initiatives, public institutions in both countries have invested significant funding in NGS following a political push for personalized medicine (National Academies of Sciences Engineering and Medicine 2016; Wierzbicki 2014). They even recently announced the launch of two large France-Quebec collaborations in this domain(Genome Québec et al. 2018). Thus, today, genomics research is performed in both jurisdictions, within a small number of publicly funded healthcare institutions. Contrary to other countries such as the Netherlands or the USA, NGS is usually not considered to be routine care, which makes it more interesting to analyse. Second, both jurisdictions are comparable on many levels. Indeed, although they have significant differences, the French and Quebec public healthcare systems are both universal. In addition, both jurisdictions share the same language, and follow the civil law legal tradition, although Quebec has a hybrid civil and common law system. Based on published literature and information from research collaborators and expert informants, we approached four teams, two in France and two in Quebec, which perform whole-exome sequencing (WES) on pediatric patients’ DNA. All Principal Investigators (PIs) approached agreed for their team to participate in the study. Two teams use WES to improve diagnosis and treatment of pediatric patients and families affected with *rare diseases (RD).* The two others use it to help pediatric patients with *refractory or relapsing cancers*, to gain understanding of their absence of response to standard treatments, and to find more effective alternative treatments. The processes involved in the clinical use of WES are hereafter referred to as clinical whole exome sequencing (CES). We collected data from interviews, participation to presentations and project documents, and analysed them using the NVivo qualitative data analysis software. Details on information sources, our data analysis methodology and our interview guide are available in Additional file 1.

## Results

### Projects motivations and rationale

Since one of our main objective was to understand the way these projects were launched and how they operated, we discussed with interviewees about the projects’ main motivations, and the rationale behind their use of WES.

#### Main motivation: helping patients

According to stakeholders interviewed in all four teams, the first and most important motivation behind the design and implementation of CES is to help patients. This is expressed explicitly:

“[…] we decided we wanted to develop this technology at the service of patients” *French Rare Disease PI.*

“[…] in the end, we always refer to how we can help the patient” *Quebec Cancer Bioinformatician.*

In both RD projects, the most important stated objective was to offer a diagnosis to patients who don’t have an “etiological diagnostic” *French RD PI,* often despite having gone through numerous clinical tests – a phenomenon described as a “diagnostic odyssey” *French Rare Disease Clinician.* CES is used to “answer a clinical question” *Quebec Rare Disease Clinician* about what is causing the symptoms of a specific individual, and obtaining this answer is described as a “success” *Quebec Rare Disease Researcher.*

In addition, stakeholders also described several positive downstream effects of offering a molecular diagnosis to RD patients, such as adapting care, preventing complications, offering new treatments or participation opportunities in clinical trials, and genetic counselling for the family.

At the collective level, the objective is to increase the team’s “diagnostic yield”, or the overall percentage of patients who obtain a diagnosis after CES testing. In the Quebec team, an additional objective was that of “demonstrating” that CES is possible, and that the team and institution is “capable” of offering this test to patients while respecting clinical standards.

In both cancer projects, CES is described as a “last chance” *French Cancer Bioinformatician* for patients who do not respond to conventional treatments, and who would otherwise be directed towards palliative care. Indeed, 20% of pediatric cancer patients still succumb from the disease. In France, CES is used in new clinical trials, which aim to evaluate the impact of CES on patients’ overall survival. Similarly, in Quebec, the CES project is described as a “feasibility study” designed to evaluate the team and institution’s capability to offer this alternative within the strict time constraint imposed by the poor survival rate of eligible pediatric cancer patients.

#### Research or clinic?

In this study, we also noticed the complex position of CES projects between research and clinical endeavors. As mentioned above, the ultimate goal of all four projects is to improve patient care. However, when asked directly if the projects were clinical or research projects, stakeholders interviewed provided a range of responses, and sometimes hesitated, demonstrating the complexity of the issue.

When describing the clinical aspects of the projects, stakeholders described their need to comply with formal processes to produce and interpret CES data. Bioinformaticians from all teams stated that they had to use tools that always give the same output from the same input, as opposed to the sorts of tools which can be used in research, where results can vary slightly at each run. Clinicians and PIs described how the interpretation process should be standardized to be able to produce a clinical report, which should include details on each step of the methodology followed. Stakeholders also described how lengthy and sometimes burdensome the reporting process can be.

Some stakeholders portrayed the research aspects of their projects positively, as a way to be more honest with patients, and avoid therapeutic misconceptions. This also allows teams to systematically collect data on the performance of the technology, which in turn could benefit future patients.

“It is clearly done in a context of clinical research. […] it is important to be able to correctly collect data on which information we have, how we use it, and to evaluate the contribution of what we do specifically.” *French Cancer PI.*

“we are pursuing the study to be able to analyse more patients because in the end we see the biases… our technical problems, and we get better over time” *Quebec Cancer Clinician.*

#### Why the exome?

Even though this question was not specifically asked in the interview guide, all stakeholders described the reasons justifying their choice for this technology, as though they wanted to convince the interviewer that this was a reasonable decision. They all seemed very accustomed to providing these reasons, indicating that they had already presented them in numerous occasions and contexts. Interviewees evoked three categories of reasons:

First, contrary to more focused methods such as gene panels, WES enables the team to examine most genes at once. Performing WES also allows teams to reanalyse “unsolved” patients’ data regularly in light of the most recent versions of variant databases and research results. Three of the four teams use “in-silico gene panels analysis” to focus their clinical analysis on a list of genes which are most likely to be clinically relevant for the patient. This list is established by gathering internal and external expertise, and data from international databases and most recent published research results. This enables patients to benefit from this collective knowledge rather than just that of their treating physician, who may order targeted genetic tests based only on his/her knowledge of the disease, which may be partial or outdated. Performing CES also allows teams to publish patients’ data into international databases, and in turn participate in increasing the knowledge-base on the genetic background of diseases, which may be useful to other patients.

A second set of reasons put forward was the wealth of published scientific evidence “proving” that this technique is clinically and economically efficient. All teams referred to specific publications(Bonafe et al. 2015; Lee et al. 2014; Saudi Mendeliome Group 2015; Yang et al. 2014; Yang et al. 2013), work done by institutions or laboratories^1^^,^^2^^,^^3^^,^^4^ or projects^5^^,^^6^^,^^7^ as elements of proof that CES, when performed with strict guidelines and quality controls, can be the best option for patients.

Finally, CES was described as “cost-effective”. Indeed, performing a series of targeted tests is more expensive than sequencing the whole exome directly. Considering the increasing demand for the test, several PIs explained that it was cheaper to develop the technology internally (at the level of the institution in France, and at the level of the province in Quebec) than to order the test elsewhere (institutions invoicing others for the test in France, or tests being performed out-of-province or in the USA in Quebec). Providing the test as a service to external clients was also described as a source of income for the institutions who offer the test early.

### Main challenges in “leveling-up”

The fact that the technology is “in transition” was made clear by members of all four teams. They expressed that the context is evolving, and that projects of this kind gradually make their way from the research to the clinical realm. When asked what the current main challenges were, teams provided a wide range of answers (see Table 1: Main challenges), some of which were previously identified in the literature, but also others which were either not identified, or not previously described in those terms.

**Table 1:** Main challenges. This table presents interviewees’ answers to the following question: “what would you say is the main challenge for clinical exome sequencing to succeed in your country/province?”

		France	Quebec
Cancer	Principal Investigator	Managing the complexity of the data and of cancer	Give targeted molecules identified through WES to patients
	Clinician	Data analysis	Data interpretation
	Bioinformatician	More rapid and efficient data analysis process	Standardized use of analysis software and pipelines
	Head of biochemistry lab	Standard clinical analysis of exome data	
Rare Diseases	Principal Investigator	Education of practitioners to genomics	Gather support from all relevant stakeholders to enable the implementation of the technology in the public healthcare system
	Clinician	Education of biologists and clinicians who participate to data analysis and interpretation	Time and availability of qualified analysis to interpret the flow of data.
	Researcher		Variants clinical interpretation
	Bioinformatician	Challenges linked to the bioinformatician profession, interdisciplinary and at crossroads between biology and computer science	Standardized bioinformatic pipeline for clinical data analysis. More investment in required storage and processing infrastructure

#### Managing the complexity of WES data

WES generates a lot of difficult-to-interpret data for each patient. Indeed, three stakeholders referred to the data stemming out of WES as ‘mostly grey’ or as situated in a ‘grey zone’, with an unclear clinical significance. Therefore, many stakeholders expressed challenges linked with the complexity of the CES process, starting from the raw fastQ file generated by the sequencer and ending in an informative clinical report.

##### Bioinformatic analysis

When describing the bioinformatic analysis, all teams described how they developed and regularly updated their pipelines. These pipelines are composed of three kinds of steps, each with their associated challenges.

I) Quality control steps, in which specific parameters are chosen to identify the subset of data that reaches the minimum level of quality for a clinical test. The issue is that although there are best practice guidelines, to date there is no formal clinical certification available for genomic tests in France and Quebec, and no collective agreement on what those minimum quality levels are.
II) Software steps, in which the data is gradually transformed from short DNA sequence reads to a list of variants which are carried by the patient. Several software packages that perform the same tasks are available, and they evolve constantly as their developers release new versions of the tools. Again, in the absence of formal standards, choosing which software to include, and when and how to update it, is a challenge.
III) Finally, in the database steps, patients’ variants (usually tens of thousands) are filtered through software which predict how they impact the resulting proteins, or through several other lists of variants that have been found in other patients^8^^,^^9^^,^^10^^,^^11^^,^^12^^,^^13^ or in a healthy population^14^^,^^15^. Here, the challenge is to choose which database to use, based on their quality, comprehensiveness, and relevance. Like software tools, databases evolve over time, and not all are available free-of-charge. Another step used by three of the four teams is that of “in-silico panel analysis”, in which they focus their analysis on a subset of genes relevant to the clinical question. These lists of genes are established by the teams and are updated regularly based on the most recent published evidence. In the context of cancer, to select actionable variants, they also consider existing drugs targeting the molecular variants, or open clinical trials in which the patient could participate. None of those steps are therefore fixed in time, and stakeholders expressed difficulties associated with the need to constantly monitor the literature and other resources in order to stay up-to-date and offer patients the best possible chance of a clinical answer. They expressed their wish that more resources would be allocated to this at the institutional level.

##### Clinical interpretation

After these automated or semi-automated steps, which can generate up to 50 to 80 variants per individual, clinicians and biologists review each “shortlisted” variation *French Rare Disease Clinician*, in order to produce the final CES report. Cases are also discussed in a group with various experts, and the final decision on what to report, reached by consensus, is signed off on by a clinician from the team before it is reported to the ordering clinician and to the patient. The most critical issue mentioned here was the time spent on each patient’s data. Indeed, some results are long and complex to interpret, because variants may have been associated with a wide variety of phenotypes, may be of incomplete penetrance, or have an effect that is less well-known. This interpretation process is described as lengthy, complex, and limited by “human capacities” *French Rare Disease Clinician*. Interestingly, several clinicians perceived this step as more critical, more ‘empirical’ and less standardized than the bioinformatics steps. They described the bioinformatics analysis as a “resolved bottleneck” *Quebec Rare Disease Clinician,* a difficulty that is “manageable” *French Cancer Biochemist*, or a process that is “well-established” *French Rare Disease Clinician* or “well-oiled” *French Cancer Biochemist*. This vision was not shared by the bioinformaticians we interviewed, who also saw their own tasks as ‘empirical’, and rather described how they felt their most important mission was to deliver a variant list which would be small enough to be “manageable” by clinicians:

“The exome covers too many genes for a human to be able to give a diagnosis on the entirety of the genome. […] And clinicians, cytogeneticists, they focus on twenty, maybe thirty genes. They have trouble focusing on more. I mean, humanly, it’s complicated. […] if you give them a list of a hundred mutations… […], clinicians don’t want it, they throw it back at your face. He will say, are you crazy, what do you want me to do with this? I want only a list of a few dozens, maximum, of genes involved in cancer, that’s it.” *French Cancer Bioinformatician.*

Regarding the question as to whether it was desirable and possible to set in place this whole process, by standards, regulations or certification, stakeholders were not all in agreement. For one team’s bioinformaticians, this was actually the most important issue:

“for our part, […] it’s just… to have first a tested and robust infrastructure, so going from a framework of research, where we have something that works, but that remains slightly blurry, to have something really very… very very structured, very well defined. Ehm... for us that is the biggest step in the short and mid-term…” *Quebec Cancer Bioinformatician*.

Although most agreed that they would benefit from more formal guidelines on how to streamline this process, some expressed that the ideal process would always depend on the specific clinical question asked. Indeed, pipelines and filtering steps are tailored to each project, each patient population, and the overall objective of the CES process. In addition, these regular updates, although burdensome to monitor, were also described as extremely beneficial in improving the efficacy of the CES process, and changing too rapidly for it to be enshrined in a law:

“The problem is that everything evolves faster than the law can, I think. It evolves very fast, new machines come out every six months. […] so if the law establishes ‘you have to use GATK version 3.3.2 for x years’ and there is a bug or a functionality that will not evolve because there is a novelty, well you’ll be in trouble. That’s the problem, it will never evolve as fast.” *French Rare Disease Bioinformatician.*

Another issue was that, although efforts are being made in this direction, it may be impossible to generate a consensus around which pipeline teams ought to use, or how to analyze the data.

#### Education

Another identified consequence of the complexity of WES data was the need for more education on clinical genomics. A wide variety of stakeholders were described by team members as needing more training on how to use and interpret genomic data, including biologists, clinicians, geneticists, and bioinformaticians. Interviewees pointed to examples of other teams who had difficulties setting up CES because of a lack of specific training on how to produce, classify and interpret the data. They even mentioned that some groups or are not aware of biases in the technology, and are not using it properly, using “wrong filters” *French Cancer Bioinformatician*. In cancer teams specifically, the need for clinicians and others to have a more realistic view of technological limitations of NGS was also highlighted as a way to avoid overselling the technology, and to manage patients’ and families’ hopes appropriately:

“Then, there is also an emotional dimension behind, where like very often in oncology and in human pathology, in oncology, we sell things like they are a solution, I sometimes end-up in situation where I’m told: “but you have to do the exome, the patient is not well, it’s the only way to cure him…” no, it’s not the only way to cure him, you mustn’t do these things, and all we will generate is information with an insufficient level of proof. And even if we generate with a sufficient level of proof, this doesn’t necessarily mean that we have a therapeutic solution to treat him”. *French Cancer Biochemist.*

Another important element was the critical importance of bioinformaticians, who represent the cornerstone of a successful implementation of CES. Their interdisciplinary training in computer science, statistics and biology is indeed necessary in order to manage the translation of raw sequencing reads into meaningful clinical information. The need to train more bioinformaticians at the national level, and to have more of them involved in teams who want to set up CES, was highlighted repeatedly.

Another category of stakeholders who were portrayed as lacking training in genomics are those in charge of technology assessment at the governmental level. Indeed, their limited knowledge in this field was seen as a barrier impeding the smooth translation of WES to a clinically approved, governmentally funded test.

#### The need to convince across the board that CES is a good idea

Another theme that emerged was the need for team members to get buy-in from a complex network of stakeholders. Indeed, establishing and standardizing the process to obtain, analyze and use genomic data in the clinic is complex, and costly in personnel and infrastructure. Therefore, many stakeholders have to be involved, and convinced that the benefits of CES are worth the effort. We have divided these stakeholders into two main categories: practitioners, and governmental stakeholders.

##### Clinicians, molecular geneticists and professional societies

First and foremost, interviewees described that clinicians should be convinced that using this test could be beneficial for their patients. When explaining why clinicians are sometimes reluctant to prescribe CES tests, interviewees talked about the “fear” *French Rare Disease PI* of incidental findings (IF) and of uncertainties associated with reporting strategies, the need for an adapted consent form, doubts about the data analysis process, and the need to be convinced that the test is more effective than more classical targeted tests. One solution provided to this issue is to involve the clinicians early-on in the project so that they have a say in how the data is reported to them, and what kind of results they will have to report to their patients.

Secondly, the community of clinical geneticists and professional societies in molecular genetics also have to reach a consensus that WES is more efficient and cost-effective than sequencing a panel of genes. This question was described as “still debated” *French Rare Disease Clinician* and causing “reluctance” *Quebec Rare Disease Researcher* from some, although this resistance was described as being on the decline. The French RD team described how, because of this controversy over the technology, some teams performed WES almost in secret:

“In the clinical framework, I think there are many people who do it but don’t dare to say it because […] it’s still debated in the geneticists’ community - should we or should we not do the exome? Should we study gene panels […] So people are led to do it anyway, and then in a grey zone diagnosis-research, they don’t announce it, it’s not clear, and above all they don’t talk about it much so it remains unclear.” *French Rare Disease Clinician.*

Especially in France, the important role of professional societies in generating guidelines on how to design consent forms, on what to include in the report and what to do with IF was described as something that could alleviate controversies around clinical genomics and convince public authorities to invest the necessary resources for responsible use of the technology. Although stakeholders complained about the absence of official French guidelines, they did not portray this as a sufficient reason not to develop the technology. Instead, they followed the guidelines they perceived as most appropriate, such as European recommendations from EuroGenTest^16^ for data analysis and interpretation, and the design of CES reports. Existing professional guidelines were also cited by interviewees, such as the ACMG guideline on reporting IFs(ACMG Board of Directors 2015), which all teams have adapted to their local context.

##### Governmental stakeholders

The other range of stakeholders referred to as critical in implementing clinical genomic testing were governmental institutions involved in healthcare.

In both France and Quebec, the Ministry of Health (MoH) was depicted as the key actor in charge of deciding if and how to implement clinical genomics. In both regions, the process of technology assessment through which that jurisdiction’s MoH has already gone to evaluate the clinical validity, clinical utility and economical sustainability of CES was described at length, with insistence on its inefficiencies. One stakeholder expressed the need to “challenge the system” *Quebec Rare Disease PI.* All teams mentioned having participated actively in the process of generating evidence to prove that CES is a valid test, but having failed to ‘convince’ the government so far. This was done by mounting specific proof-of-concept or medico-economic studies, and by submitting results to the relevant decision-makers. All project leaders described similar frustrations linked to the authorities’ inability to recognize the clinical and economic benefits of WES, even though they and other teams around the world had produced an increasing amount of scientific evidence:

“we hit a wall” *Quebec Cancer PI.*

“we are fighting since 2012 to make them understand that high throughput is now, not in ten years” *French Rare Disease PI.*

“I think there may also not be enough solid data in the literature, or in what we do in our research to convince them [the government] maybe” *Quebec Cancer Clinicia*n.

They therefore expressed their conviction that in addition to solid scientific and economic evidence, the implementation of WES could not be done without clear political will from the highest levels of government. Indeed, there was consensus that implementing CES entailed a clear commitment of the state to personalized medicine, and could only be done at the national level with a clear country or province-wide organization of services, significant investments in sequencing and data storage infrastructures, and in training of professionals.

“[…] who does what, should there be one, two, three, four platforms? […] Who will capture the sequences, who will return results, depending on the platform how far do we go, should they return raw results, will existing diagnostic labs analyse the data… there is a whole organisation, I would say… biological, to be thought through. With quite notable territorial inequality, I think in terms of training of biologists to interpret the data” *French Rare Disease PI.*

Importantly, actors highlighted a need to reach a broad consensus on how to frame the use of WES, namely determining which patients should be offered the test, which doctors should be allowed to order the test, where and how the data should be sequenced, stored and analysed, and finally who should report clinical results and how. The ‘finish line’ would be for CES to be offered as a standard test for specific patients, with a formal price quotation, reimbursed directly though the public healthcare system.

“*French Rare Disease PI*: And the final success would be that it is paid by the public authorities.”

*G.B.*: The reimbursement.

“*French Rare Disease PI*: Yes, exactly. It would really be the final success. This means that patients with a genetic disease could benefit from this technique in diagnosis, and reimbursed, I mean covered. So covered, how do I say this? Not necessarily 100% from the Social Security, there could be a part covered by private insurance, why not? But that there could be a coverage, really, by the health system.”

At the time when interviews were performed, both the French and the Quebec governments were consulting experts on how to implement those tests. We got a sense from all teams that this political will was emerging and that things could move soon in this domain.

### What will the future look like?

When asked what the future of clinical genomics would look like in the next 5 years, stakeholders depicted many changes, illustrating how fast they believed the field is moving. (See Table 2: What will change in 5 years?)

**Table 2:** What will change in 5 years? This table presents interviewees’ answers to the following question: “what do you think will change in five years?”

		France	Quebec
Cancer	Principal Investigator	We will know more on the biology of cancers.	Genomics will be integrated in clinical practice, with a hybrid clinical and research mission.
	Clinician	We will have a standardized data analysis process.	WES will be approved for use in the clinic, and more will be understood about the biology of cancer.
	Bioinformatician	Technology will be available across the territory.	WES and transcriptome will be used in the clinic, and all patients will be sequenced.
	Head of biochemistry lab	Technology will be stable and costs will go down	
Rare Diseases	Principal Investigator	WGS will be used instead of WES, and used in rare diseases, cancers and common diseases.	Only one genetic test will be used, WGS, as long as it becomes cheaper than WES and targeted tests.
	Clinician	Genomics will be used for rare diseases, cancers and common diseases.	WES will be a formal clinical test offered with the appropriate resources, and will be applied in more diseases.
	Researcher		WES will be implemented in the clinic, and WGS will be in the process of evaluation for the clinic.
	Bioinformatician	WGS will be used in the clinic.	The process of sequencing and analysis will be standardized throughout the province.

#### Technological developments

First, a number of interviewees talked about technological developments which they are either certain, or hope, will occur within the next 5 years. Some mentioned the necessary improvement of the “cost and performance” *French Cancer Biochemist* of WES, such as the percentage of exons captured and sequenced at sufficient coverage.

Another important theme was that of the transition from WES to WGS. Indeed, WGS not only enables the analysis of all genes at an equivalent coverage level, but also uncovers large-scale rearrangements, small and large copy-number variants, and intergenic regions. The main difficulty raised about WGS is the cost of storage and computing infrastructures needed to store and process the data. There was a general consensus that in the clinic, the analysis would be focused on the coding regions of the genome first, but that data should be shared and used in research, and should remain accessible for regular clinical reanalysis. In cancer, where researchers and clinicians are confronted by highly complex tumor genomes, stakeholders also described other promising technological developments, like circulating tumor DNA or immunotherapy. Several interviewees therefore described WES as “a first step among others” in clinical genomics:

“The exome is absolutely not an end in itself, but a step, in fact, at the level of genomic technologies, towards tests which will eventually be better but that, in the context… in the present context, is the best we can offer patients within the clinical structure of the hospital” *Quebec Rare Disease Clinician.*

#### Transition to clinical standards

Echoing the issues raised in 2 – Main challenges in “leveling up”, most stakeholders also expressed their belief that within 5 years, WES will probably be a standard clinical test, offered through the public healthcare system to all patients who need it. There will be no “need to do it in the research context” *Quebec Cancer Bioinformatician*, and data interpretation will be rendered easier by advances in research and increased data sharing. Governments will have taken decisions as to which patients to offer the test to, possibly through the setup of “pilot projects” *French Rare Disease PI, Quebec Rare Disease PI*. The production of sequences will be organized throughout the territory, through certified platforms. Analysis pipelines will also have been standardized, and the legislative framework for the storage, sharing and security of patients’ WES data, including IFs, will have been established. There will also have been significant progress in the training of practitioners and biologists to use and interpret genomics data to improve patient care. Access to the technology will therefore be organized and democratized.

“I hope I’m not wrong by thinking that in five years, at least the part that we call now ‘clinical’, this part will really be a clinical test in due form, which means covered by the government, subject to specific turnaround time but also to resources, to weighted values at the level of the institution, which should in fact help so that, for instance, the time of the analyst would be easier to match the analysis volume.” *Quebec Rare Disease Clinician.*

In France, however, one PI expressed doubts that the test would be reimbursed by the healthcare system within just 5 years:

“In terms of reimbursement, etc.… coverage by the social security and all, I think we won’t be there at all in 5 years. No, we have to be lucid… I think it’s wishful thinking. But if already we can put in place a system where it stays within nomenclature and that at least some institutions... [hesitation] I think already it would be a huge step.” *French Rare Disease PI.*

#### Broaden the access

All teams agreed that cancer and RD were the two domains in which genomic tests would be the most useful in the short term, but some mentioned that this could eventually be useful for patients with common diseases such as diabetes, and for pharmacogenomic testing. In cancer, stakeholders described their hope that all or most patients would be sequenced at diagnosis, and not only when they relapse or after their first unsuccessful treatment, although not all were confident this would be the case within only 5 years. In France in particular, interviewees described how important it was to resolve the current territorial inequality in access to WES. Currently, a RD or cancer patient may not be offered CES, either because no research team has put it in place so far in the healthcare institution where she is treated, because the institution has not invested in sequencing technologies, or because they don’t have qualified personnel in house to interpret the data. He/she may then be forced to travel to another region to access the test, which is a significant issue for patients with low resources or whose condition limits their mobility. It was therefore highlighted that a national organisation for genomic sequencing would allow personalized medicine to be established in France while respecting important French values.

“*French Rare Disease PI:* So that’s the ultimate goal, it’s to manage that the French organisation would allow for patients who don’t have a diagnostic and who are at high suspicion of having a genetic disease to have access to this technology.”

*GB:* Whatever their reference center is… or wherever they are in the territory?

*French Rare Disease PI:* Well if we want to go back to the ‘Franco-French’ theme, that’s the French idea, it’s access to care for all, and at a minimal cost for the patient… so I won’t say free because patients are… unfortunately not everything is free, but at the lowest cost for patients. And that is the French vision of health ».

## Discussion

### Quebec and France

In both regions, the ‘political will’ which was described by interviewees as indispensable is now present, and both governments have, while data collection for this study was taking place, taken steps to move forward with clinical genomics. In June 2016, the president of the French National Alliance for Life Sciences and Health (Aviesan), published a publicly available report(Lévy 2016) paving the way for medical genomics to be implemented in France by 2025. The two first national sequencing platforms started to be active in the fall of 2017^17^. In 2015, the Quebec Minister of Health sent a call for proposals to all seven supra-regional university hospitals for establishing a clinical genomic platform. It has since received proposals but still not published its final decision, which could mean that although the government acknowledged that CES is needed, this is not ‘the political priority’ at the moment, or that they are proceeding very cautiously.

### Rare diseases and cancer

Overall, although all four projects are operating at the crossroads between research and clinical practice, cancer projects seem less advanced than RD projects on the translational path. Indeed, RD team members cited numerous publications and collective experiences demonstrating that CES does improve the diagnostic yield of patients with undiagnosed Rare Diseases, and could also contribute to the improvement of treatments in the future. However in cancer, the objective of CES is to contribute to increasing patients’ overall survival rate by providing targeted treatments. However, team members insisted on the benefits of CES in increasing knowledge and understanding of the disease, and in CES findings providing avenues for future clinical trials, rather than describing CES as currently able to ‘save patients’. A number of issues discussed within cancer teams were unique to this context, including, the need to engage with the pharmaceutical industry in order to broaden the scope of trials design and the number of treatments offered to pediatric patients. The time sensitivity and the need to provide CES results as fast as possible also seems much more critical in a context where cancer patients will potentially pass away within a few weeks, rather than in the case of patients who have already been waiting for a diagnosis for several years. Therefore, cancer teams also discussed the need to involve and obtain buy-in from a chain of specialists in the process, from laboratory technicians to surgeons, pathologists, and oncologists, in order to orchestrate the whole CES procedure fast enough to provide potentially actionable results in time. Finally, cancer DNA is much more complex and challenging to extract, isolate and analyse(Bertier et al. 2016a) than germline DNA.

In both contexts though, teams described the need to perform the CES test early, as a first-tier test in RD to avoid multiple unsuccessful targeted tests, or at diagnosis instead of after relapse in cancer, in order to have a view of the disease mutational landscape before selecting first-line treatment.

### Relevance for policy

By using a case study analysis model, which enables the researcher to build a relationship of trust with stakeholders, and to have a comprehensive view of the way they operate through multiple information sources, we were able to gather information from the ground on elements that are difficult to find otherwise. Indeed, although examples of successful CES implementation projects are becoming more common in the literature, to our knowledge no study has been published so far which identified other ‘non-scientific’ elements which can impact the success of CES projects. We were indeed able to describe the complexity of logistical, political and interpersonal factors that need to be taken into account, in addition to financial and scientific matters, in order to offer CES to patients at the national level. We strongly believe that results from this and other observational studies could be used to support the development of policies grounded in evidence, which are more likely to be implemented with ease. For instance, we observed a consensus on the importance of bioinformaticians, and of training more stakeholders in genetics for CES implementation to succeed.

### Limitations

Since the start of data collection, major changes have occurred in the legal and regulatory landscape which will impact the clinical use of sequencing in the near-future. For instance, in FR, in addition to the Aviesan report(Lévy 2016), application decrees^18^ were published in 2016 on the law on human research (or Jardé law), which will have an impact on the practice of genomics(Levy et al. 2017; Mamzer 2017). In addition, a large public consultation on the revision of the bioethics law was launched in March 2018^19^, which notably questions citizen on the use of genetic testing and genomic medicine^20^. Another challenging element for data analysis is that teams operated within a complex network of rules and regulations, both at the institutional, regional, national and provincial levels. Relying on actors on the ground is a benefit of the case studies approach, but it can also be a limitation, since their answers may be biased toward advocating for the importance of the projects they developed. Because of the complexity of the method, we were not able to include more than four teams in the study, but other groups may have provided other interesting perspectives on the matter.

## Conclusions

In this study, we documented the work, challenges, motivations and vision of professionals from Quebec and France who use NGS to inform patient care. Although WES is not a validated clinical test yet, there are teams who do use this technology in the clinic. The CES projects we explored stand at the crossroads of research and the clinic, and display characteristics of both domains, rendering the identification of their appropriate legal and policy framework extremely complex. Implementing CES at the level of these teams required significant financial, scientific, infrastructural, logistical, and inter-personal efforts to streamline the numerous steps required to extract, analyse and interpret CES data. Implementing this technology efficiently at the national level will require similar efforts to be performed at a much greater scale and in a centralized manner, which cannot be done without strong political will at the highest levels of government. Indeed, managing the extreme complexity of CES process and data will require the involvement, buy-in, education and training of a complex network of stakeholders including practitioners and public authorities’ representatives. This political will is present in France and also, at some level, in Quebec. Results of this study could be used among other evidence by policy makers in both regions to establish national personalized medicine programs. However, more research is needed on the legal and regulatory frameworks specifically applicable in both regions, taking the specificities of each healthcare system, legal landscape, and population structure into consideration.

## Additional file

Additional file 1: Details on data collection and analysis methodology. (DOCX 30 kb)

## Abbreviations

ACMG: American College of Medical Genetics and Genomics
CCMG: Canadian College of Medical Geneticists
CES: Clinical exome sequencing
ISO: International Standards Organisation
PI: Principal investigator or group leader
RD: Rare disease(s)
WES: Whole-exome sequencing

## Footnotes

1 Baylor Medical Genetics Laboratories, https://www.bcm.edu/research/medical-genetics-labs/test_detail.cfm?testcode=1500 Accessed 10 July 2018

2 UCLA Clinical Genomics Center, http://pathology.ucla.edu/genomics Accessed 10 July 2018

3 Genome Diagnostics Nijmegen, http://www.genomediagnosticsnijmegen.nl/index.php/en/services/exome-sequencing-diagnostics Accessed 10 July 2018

4 The Terry Fox Research Institute, http://www.tfri.ca/ Accessed 10 July 2018

5 The Deciphering Developmental Disorders Project, https://www.ddduk.org/ Accessed 10 July 2018

6 The Care for Rare Project, http://care4rare.ca/ Accessed 10 July 2018

7 The Kids Cancer Sequencing Program, KiCKS, https://kicsprogram.com/ Accessed 10 July 2018

8 ClinVar, https://www.ncbi.nlm.nih.gov/clinvar/ Accessed 10 July 2018

9 The Human Gene Mutation Database, http://www.hgmd.cf.ac.uk/ Accessed 10 July 2018

10 Orphanet, http://www.orpha.net/consor/cgi-bin/index.php Accessed 10 July 2018

11 The Cancer Genome Atlas, https://cancergenome.nih.gov/ Accessed 10 July 2018

12 My Cancer Genome, https://www.mycancergenome.org/ Accessed 10 July 2018

13 FoundationOne, https://www.foundationmedicine.com/genomic-testing/foundation-one Accessed 10 July 2018

14 The Exome Variant Server, http://evs.gs.washington.edu/EVS/ Accessed 10 July 2018

15 The Exome Aggregation Consortium (ExAC) http://exac.broadinstitute.org/ Accessed 10 July 2018

16 The EuroGentest project, http://www.eurogentest.org/index.php?id=160 Accessed 10 July 2018

17 Announcement of the two first Genome Sequencing plafroms from the France Genomic Medicine 2025 plan, https://aviesan.fr/aviesan/accueil/toute-l-actualite/lancement-des-deux-premieres-plateformes-du-plan-france-medecine-genomique-2025 Accessed 10 July 2018

18 Official application decree from the Jardé law, Décret no 2016–1537 du 16 novembre 2016 relatif aux recherches impliquant la personne humaine, http://www.dm-experts.fr/wp-content/uploads/2016/11/2016-11-17_Decret_application_loi_Jarde.pdf Accessed 10 July 2018

19 Etats Généraux de la bioéthique, https://etatsgenerauxdelabioethique.fr/ Accessed 10 July 2018

20 Examens génétiques et médecine génomique, Etats Généraux de la bioéthique, https://etatsgenerauxdelabioethique.fr/project/genetique-et-genomique/presentation/presentation-7 Accessed 10 July 2018
