# Supplementary material for: Clinical exome sequencing in France and Quebec: what are the challenges? What does the future hold?
Source: Life Sci Soc Policy. 2018 Aug 1;14:17. doi: 10.1186/s40504-018-0081-2 (PMC6068066; doi:10.1186/s40504-018-0081-2)
Supplement: Supplementary file 1 — Details on data collection and analysis methodology. (DOCX 30 kb) [file 40504_2018_81_MOESM1_ESM.docx]

# Additional file: Details on data collection and analysis methodology

## Information sources

Data collection started in November 2015 and was completed in July 2017. The case studies are based on three main sources of information: interviews, project presentations and project documentation.

In each case, GB conducted interviews with at least three stakeholders, namely: the PIs leading the project, a bioinformatician who analyses patients’ WES data, and a clinical geneticist or a clinician, who produces the WES report and communicates results to patients or their referring clinician. We designed partly overlapping interview guides (see interview guide below) for each type of stakeholder for one hour long semi-structured interviews. A total of 23 interviews were completed and transcribed verbatim by a professional transcriber. 22 interviews were conducted in French and 1 in English.

GB attended a total of 10 project meetings in which project strategies or project results were discussed.

Finally, we collected a variety of documents which provide a detailed overview of the projects’ design, data management plan and results. These include: grant proposals submitted, PowerPoint presentations, consent forms, CES analysis reports used to inform doctors and patients of the test results, and bioinformatics analysis pipelines.

## Analysis strategy

The information collected in each case was coded in NVivo. First, a novel thematic tree was generated directly from the data, using an inductive method^[[1]](#footnote-1)^. Second, this thematic tree was compared to one generated from previous publications^[[2]](#footnote-2)^^[[3]](#footnote-3)^, following the deductive analysis method^[[4]](#footnote-4)^. We also explored similarities and differences across French and Quebec cases, across case types, and across interviewed stakeholders. Inter-rater validity was obtained in two ways: first, the thematic tree obtained through the deductive analysis was discussed with two independent researchers who are experienced with qualitative data analysis and with NVivo, but were not involved in either the project design or the data collection. Second, the latter researcher co-coded one interview. All differing codes were discussed with the lead author (GB) and the thematic tree was adapted based on consensus coding.

## Interview Guide

| **Questions** | **PI** | **Clinician** | **Bioinformatician** |
| --- | --- | --- | --- |
| **Introduction** | What is your current position, and how long have you occupied it? | | |
|  | Is WES used in your institution for research or for clinical purposes | | |
| **The project** | Describe where the idea of the project comes from | Since when/why/how are you involved in the project? | |
|  | Describe the project rationale | Please provide a description of the project from your perspective | |
|  | How is the project funded? |  |  |
|  | How is the project advancement monitored? |  |  |
| **Data production,**  **analysis,**  **reporting** | Could you describe briefly how WES data is produced-analysed-reported | Walk me through a typical patient referral process | Walk me through a typical data analysis process |
|  | Who is in charge of data analysis? | Describe briefly if/how you discuss results with the project team, and how you communicate results to the patients and their families | Who finally decides which results are reported? |
|  | Are you personally involved in data interpretation? |  | Who do you report the results to? |
|  | What is a typical timeline between reception of the raw data and reporting? | | |
|  | Are WES data reused in research or reanalysed for further patient care? | | |
| **Guidelines** | What forms, protocols, guidelines (internal or external) did you have to follow to set up the use of WES data in the project | What forms, protocols, guidelines (internal or external) do you follow to include patients in the project and to report the data to patients and their families? | What forms, protocols, guidelines (internal or external) did you have to follow to set up the use of WES data in the project |
|  | What is your opinion on these guidelines? | | |
| **The present** | What is the current progress of the project? | | |
|  | What is main challenge of the project, and why? | | |
|  | What would be the main indicator of the success of the project? How/when do you think this will happen? | | |
|  | Should WES be introduced in routine clinical care for cancer/rare disease patients in France/Quebec? | | |
| **The future** | What do you expect will change in the next five years? | | |

1. Mucchielli A: Dictionnaire des méthodes qualitatives en sciences humaines. ed 2 Paris, Armand Colin, 2004. [↑](#footnote-ref-1)
2. Bertier G, Hétu M, Joly Y: Unsolved challenges of clinical whole-exome sequencing: a systematic literature review of end-users’ views. BMC Med Genomics 2016;9:52. [↑](#footnote-ref-2)
3. Bertier G, Sénécal K, Borry P, Vears DF: Unsolved challenges in pediatric whole-exome sequencing: A literature analysis. Crit Rev Clin Lab Sci 2017;54:134–142. [↑](#footnote-ref-3)
4. Elo S, Kyngäs H: The qualitative content analysis process. J Adv Nurs 2008;62:107–15. [↑](#footnote-ref-4)
